# Nephrologists’ experiences with patient participation when long-term dialysis is required

**DOI:** 10.1186/s12882-021-02261-w

**Published:** 2021-02-16

**Authors:** Tone Andersen-Hollekim, Bodil J. Landstad, Marit Solbjør, Marit Kvangarsnes, Torstein Hole

**Affiliations:** 1 Department of Circulation and Medical Imaging, Faculty of Medicine and Health Science, Norwegian University of Science and Technology, Trondheim, Norway; 2 Department of Health Sciences, Mid Sweden University, Östersund, Sweden; 3 Nord-Trøndelag Hospital Trust, Levanger Hospital, Levanger, Norway; 4 Department of Public Health and Nursing, Faculty of Medicine and Health Science, Norwegian University of Science and Technology, Trondheim, Norway; 5 Department of Health Sciences, Faculty of Medicine and Health Science, Norwegian University of Science and Technology, Ålesund, Norway

**Keywords:** Patient participation, End-stage renal disease, Nephrologists, Dialysis care

## Abstract

**Background:**

For individuals in need of dialysis, patient participation is important when determining care goals and in decision making regarding dialysis modality. Nephrologists hold a key role in delivering evidence-based healthcare that integrates patient preferences and values throughout the trajectory, and their experiences with patient participation are important for improving health care. The aim of this study was to explore nephrologists’ experiences with patient participation in different phases of the end-stage renal disease trajectory for working-age individuals who require dialysis.

**Methods:**

This explorative study comprised interviews with ten nephrologists from four different dialysis units in Central Norway. We analysed the interviews by applying an interpretive phenomenological approach.

**Results:**

Nephrologists had varied experiences with patient participation throughout the different phases of the treatment trajectory. During decision making on the dialysis modality, nephrologists emphasised patients’ choices in two approaches. In the first approach, they expected patients to choose the modality based on the provided information, which could be actively steered. In the second approach, they recognised the patients’ values and lifestyle preferences through shared decision-making. Within hospital haemodialysis, nephrologists considered patients’ self-care activities equivalent to patient participation, seeing self-care as a source of patient empowerment. They identified divergent patient–professional values and organisational structures as barriers to patient participation.

**Conclusion:**

Our study shows that nephrologists have different approaches to patient participation in different phases of the end-stage renal disease trajectory. Individual understanding as well as organisational structures are important factors to address to increase patient participation in end-stage renal disease care. Shared decision making, in which patient values are balanced against biomedical treatment targets, allows for mutual agreement between patients and healthcare professionals concerning medical plans and minimises the potential for patient–professional tensions.

## Background

The patient’s position has been increasingly strengthened throughout the recent decades, making patient participation an essential part of medical treatment and care for people with chronic illness [1, 2]. In end-stage renal disease (ESRD) care, nephrologists have a central role in delivering evidence-based healthcare that integrates patients’ preferences and values [3]. They prescribe and monitor dialysis treatment, collaborate closely with other healthcare disciplines, and learn about their patients’ lifestyles and preferences through long-term medical follow-up [4]. Hence, nephrologists are positioned to optimise patient participation throughout the patients’ treatment trajectory. However, their role in facilitating patient participation is scarcely addressed. In this study, we explored nephrologists’ experiences with patient participation in different phases of the ESRD trajectory.

Loss of kidney function could be acute or chronic [5]. Even though both may require dialysis, acute renal injury comes with the possibility of remission, while chronic kidney disease (CKD) progressing into end stage requires long-term dialysis for the patient to survive [6]. According to the Norwegian Renal Registry [7], patients not preemptively transplanted had been in dialysis for a median of 1.9 years (mean 2.3), ranging from 1 week to 13.7 years.

While patient participation is recognised as a key factor in improving the delivery and quality of health services [8], practical implementation has proven difficult [9]. Barriers are related to time constraints, role expectations and power ineqities, insufficient training and lack of a common conceptual understanding, among other factors [1, 9–11]. The term patient participation could be used interchangeably with shared decision-making, an approach especially suitable when more than one applicable treatment option exists [12].

For people entering long-term dialysis, patient participation is related to pre-dialytic treatment decisions, including the choice of dialysis modality, or considerations when adapting dialysis treatment to everyday life, such as determining the goals of care [6, 13]. However, previous research has identified several deficiencies regarding patient participation along the ESRD trajectory, in which patients have reported suboptimal satisfaction with for instance the process of selecting a dialysis modality [14, 15].

Nephrology practices may be influenced by how nephrologists perceive their role as a physician, as found by Ladin et al. [16]. In this study, practices ranged from protective paternalism to the more patient-centred informative and interpretive and approaches. In the informative approach, nephrologists viewed themselves as patients’educator and emphasised patient autonomy in decisions. In the interpretive approach they emphasisised patient goals and values through shared decision-making [16]. Nephrologists who aim to engage their patients may experience patient participation difficult to achieve in a field driven by biomedical and measurable targets [3, 6, 17]. Their efforts to involve patients may be limited by ambiguities about how to manage critical comorbidities and broader quality of life outcomes within a technically demanding setting [3, 17].

Divergent values between nephrologists and their patients may cause tensions or conflicts in ESRD care, especially in hospital haemodialysis [6, 18]. For instance, patients who prioritise personal wellbeing and maintaining their life outside the dialysis ward [6, 19] may value biomedical targets, important for nephrologists in evaluating and adjusting dialysis treatment, differently. Similarly, divergent communication styles can create tensions, for instance when nephrologists practise a standardised, ‘one size fits all’ communication style, while patients prefer a personalised approach [18]. Although not unique for ERSD care, the imbalanced patient–professional power represents a challenge to patient participation. By tradition, physicians may consider themselves as someone who take responsibility, determine treatment options, and are trusted by patients [20]. Hence, they could view patients who participate through questioning their treatment or requesting additional information as being critical or mistrusting [20].

Although previous studies have explored nephrologists’ experiences with patient participation in ESRD, these studies essentially focus on elderly patients [16] or on patients regardless of age [3] and often involve end-of-life discussions [21, 22]. At a stage in life where education, employment and personal relationships are pivotal, entering dialysis may limit the individual’s life choices and put patient values at stake. Hence, patient participation may be especially pressing in ESRD care [23]. Little is known about how nephrologists experience patient participation for working age individuals who require dialysis. Examining nephrologists’ experiences with patient participation may contribute to identify areas for improving quality of care throughout the ESRD trajectory.

## Methods

### Aim of study

The aim of this study was to explore nephrologists’ experiences with patient participation in different phases of the end-stage renal disease trajectory for working-age individuals who require dialysis.

### Design

This explorative study comprised interviews with ten nephrologists from different dialysis units in Central Norway. We analysed the interviews by applying an interpretive phenomenological approach [24].

### Recruitment and study participants

We applied a purposive sampling strategy [24] that included physicians and nephrologists who treat working-age adults undergoing hospital haemodialysis. We carried out recruitment at four dialysis units in Central Norway. Inclusion criteria were as follows: Norwegian-speaking nephrologists or physicians of various ages and genders with a minimum of one-year experience in dialysis care. Out of 13 invited participants, nine nephrologists and one nephrology trainee participated, four of whom were women. The nephrologists had experiences with both dialytic and non-dialytic CKD/ESRD patients. Their clinical nephrology experience varied between 5 and 20 years. The reason for non-recruitment was a lack of response to the letter of invitation. We refer to all participants as nephrologists in the following paragraphs.

### Data collection

The first author conducted face-to-face interviews in November 2019–May 2020. Based on previous research and the aim of the study, we applied a semi-structured interview guide (Table 1) related to patient participation in different phases of the ESRD trajectory. We conducted the interviews either in a sheltered area in each nephrologist’s workplace or via internet video calls. Each interview lasted between 32 and 86 min, was audio recorded and then transcribed verbatim. The interviews provided rich and diverse data. With data being repetitive, we considered saturation accomplished [24] after ten interviews.

**Table 1:** Semi-structured interview guide

Questions	
How do you prepare your patients for dialysis? How is the decision about dialysis modality made? How do you involve patients and next of kin in this decision? What do you consider important for patients to know before they commence dialysis? How can patients undergoing hospital haemodialysis participate? How would you describe the term patient participation? How do you consider nephrologists’ role in patient participation? How do you promote patient participation in your clinical work? Is there anything else you would like to convey?	

### Analysis

We approached data by using interpretative phenomenological analysis [24] that involved five key stages: familiarisation, coding, theme development, defining themes and reporting. First, we read the transcripts to get an overall impression of the data. We then inductively identified meaning units in each interview. These meaning units were coded by connecting key words to the phrases used by the interviewees [24]. Next, we clustered our coding by content, that is, we grouped together codes with similar meanings. These codes formed the foundation for the development of themes. We based theme development on two different phases of the ESRD trajectory. The first phase was related to the dialysis commencement, in which patient participation was associated with choosing dialysis modality. This choice involved hospital haemodialysis or home treatment. The second phase was related to patient participation in hospital haemodialysis. We performed the analysis in an iterative process in which we continuously rechecked development of subthemes with the transcripts. We looked for patterns within the data as well as diversity and contradictions that broke these patterns. All authors participated in defining themes and agreed upon the final themes presented. We provide selected quotations to underpin each theme. Table 2 exemplifies the development of themes.

**Table 2:** Analytical process from text segments to themes

Trajectory phase	Quotes	Sub-themes	Themes
Patient participation when dialysis is initiated	*As a rule, we let patients choose the modality they want. Of course, you can try to influence them a bit towards the direction you think wise. If people are active, we promote home-based treatment. However, the patients themselves should make the final choice.* (Participant G) *Laypeople don’t usually know anything about kidney failure and dialysis, or the difference between HD and PD – it all depends on what information they get… and we think it’s okay to start with PD…so I would say it’s a slightly steered choice.* (Participant H) *We present peritoneal dialysis in a slightly more positive way than haemodialysis. In this county, we have less home dialysis than recommended. Thus, we may be pushing the patients a little towards home treatment, to meet the policy goals.* (Participant D)	A slightly steered choice	The challenge of guiding treatment choices
	*I must become familiar with patients’ daily lives to the extent that is possible. I then inform about the different options. I would say it’s sort of a process, a dialogue, where I challenge the patients with questions to get to know their priorities.* (Participant I) *I ask about the situation at home. Whether they have kids and whether they want to continue work. And if there are other things that are important to them. And then it’s important to get them well informed about both the dialysis modalities.* (Participant A)	A shared decision	
Patient participation in haemodialysis	*As a haemodialysis patient, you can of course participate in your own treatment. You can learn to cannulate the fistula, to set up the machine and to a certain extent run the dialysis.* (Participant D) *They must be allowed to make informed choices. They must know why we do things and why they get this treatment, the point of coming here so frequently, the point of the medications.* (Participant F)	Participation through self-management	Negotiating patient participation within a professional frame
	*We don’t feel that all of them have enough knowledge about the treatment to have an opinion about it* [the fluid removal], *so no, we don’t always discuss it with them.* (Participant F) *Dialysis isn’t a pre-set treatment impossible to adjust to individual needs… many are unaware of that... but dialysis is about so much more than just prescribing a time-scaled treatment with this or that dialysate solution and this or that filter* [dialyzer membrane]. (Participant I)	Negotiated values	
	*You want to provide flexibility, but it is not always possible when you don’t have enough machines and there are cutbacks in addition and - it just makes it difficult.* (Participant A) *If you are concerned with people and your patients, you should be concerned with patient participation as well. However, it takes more of your time. If you just decide on behalf of the patients, you get things done faster.* (Participant G)	Hindrances for patient participation	

### Ethical considerations

The study was approved by the Norwegian Centre for Research Data (case number 702797). We obtained written informed consent from all participants. De-identified data was stored on a password-secured server provided by the hospital trust. To ensure confidentiality, we replaced the nephrologists’ names, ages and gender with Participant A, B, C and so on.

## Results

We present the experiences of ten nephrologists with patient participation in different phases of the ESRD trajectory based on the following themes and subthemes: Theme 1. The challenge of guiding treatment choices*: A slightly steered choice* and *A shared decision;* and Theme 2. Negotiating patient participation within a professional frame: *Participation through self-management, Negotiated values* and *Hindrances for patient participation.* The first theme relates to patient participation in the choice of dialysis modality, while the second theme relates to patient participation in haemodialysis.

### Theme 1. The challenge of guiding treatment choices

Nephrologists focused on pre-emptive transplantations for patients of working age. Such transplantations were not always accessible, leading patients to the choice of dialysis modality. In this context, we found two approaches to patient participation; informed choice and shared decision-making. In the former approach, nephrologists expected patients to choose their modality based on information provided by healthcare professionals. In the latter approach, they actively included patient values and lifestyle preferences in the modality choice. The two approaches existed simultaneously between and within the nephrologists, without a distinct difference.

#### A slightly steered choice

Consensus existed among nephrologists that patients should make the modality decision themselves or in collaboration with their families. Thorough information was emphasised as a means of enabling patients to make a choice*.* Information was provided by nephrologists, but also by dialysis nurses and through pre-dialytic education programmes. After providing information, the nephrologists commonly encouraged patients to *go home and think it over* (Participant B) before deciding. They equalled this approach to informed choice. However, there was a duality in the decision-making process. On the one hand, nephrologists emphasised individual patient choice. On the other hand, they influenced the decision-making by advocating certain treatments.

*As a rule, we let patients choose the modality they want. Of course, you can try to influence them a bit towards the direction you think wise. If people are active, we promote home-based treatment. However, the patients themselves should make the final choice.* (Participant G).

The nephrologists considered time important to allow patients to prepare for the necessity of dialysis. Moreover, thorough information about haemodialysis (HD) and peritoneal dialysis (PD) was seen vital in the decision-making process. Their way of presenting the information could direct patients towards a specific treatment modality. Nephrologists generally preferred PD as a first choice. Some suggested that people of working age should take care of their own treatment, if capable. Additionally, policy goals and medical guidelines led them to weight their presentation of treatment modalities in favour of PD.

*Laypeople don’t usually know anything about kidney failure and dialysis, or the difference between HD and PD —it all depends on what information they get… and we think it’s okay to start with PD…so I would say it's a slightly steered choice.* (Participant H).

*We present peritoneal dialysis in a slightly more positive way than haemodialysis. In this county, we have less home dialysis than recommended. Thus, we may be pushing the patients a little towards home treatment, to meet the policy goals.* (Participant D).

Although a PD-first approach was favoured, factors such as traditions and staff resources often moved patients in the direction of hospital haemodialysis. The nephrologists considered the process of initiating patients in hospital haemodialysis as well integrated, requiring less work by the staff compared to home-based treatment.

Many nephrologists found it difficult to provide patients with the complete picture of what the different modalities implied for the individual. The complexity of modality decision was emphasised through statements such as: *You don’t know what you have agreed to until you have started* (Participant B), indicating that patients had to physically undergo treatment in order to fully understand it. According to the nephrologists, patients often considered hospital haemodialysis a manageable treatment option, without being aware of the intensity of treatment, nor how it would come to influence their lives. Nephrologists could be reluctant to emphasise such consequences, as they considered it would increase patients’ burden of treatment.

The nephrologists suggested that timing the modality decision against the ESRD progression could be challenging. They considered biochemical parameters and individual uremic tolerance basis when initiating patients in dialysis. To reach preparing for dialysis, they expected patients to decide on modality in ample time ahead of parameters rising. Patients who *could not make up their minds* (Participant F) could be held responsible for postponements of the modality decision, leading to unwanted acute dialysis.

#### A shared decision

In the second approach, nephrologists considered themselves as supervisors, guiding patients towards a choice through dialogue. Nephrologists who applied this approach emphasised spending time to learn about patients’ work situations and family lives as well as their individual preferences and values. Thus, they were able to consider the decision from a holistic perspective.

*I must become familiar with patients’ daily lives to the extent that is possible. I then inform about the different options. I would say it’s sort of a process, a dialogue, where I challenge the patients with questions to get to know their priorities.* (Participant I).

The nephrologists recognised that the different modalities would affect patients’ lives with divergent intensity. Hence, they considered individual circumstances, such as whether the patient was a single parent or wanted to continue their employment, in order to select the treatment that optimally fitted the patient.

*I ask about the situation at home. Whether they have kids and whether they want to continue work. And if there are other things that are important to them. And then it’s important to get them well informed about both the dialysis modalities.* (Participant A).

According to these nephrologists, achieving shared decision-making required sensitivity to patients’ unspoken issues as well as those explicitly expressed. Additionally, nephrologists acknowledged patients’ insecurity when facing the area of medicine. Time was emphasised. They suggested that patients who had reached the final decision themselves would adapt to their treatment more easily, thereby achieving better treatment results.

### Theme 2. Negotiating patient participation within a professional frame

Within hospital haemodialysis, the nephrologists associated patient participation with self-management, for instance the performance of hands-on self-care tasks. Tensions occurring from divergent patient–professional values as well as organisational structures complicated patient participation.

#### Participation through self-management

The nephrologists recognised that hospital haemodialysis required rigid adaptation, which could provide patients with a passive role. Engaging patients in their care was considered important, as a way of making patients responsible for their own health. This could implicate training them in hands-on activities such as self-cannulating. Eventually, they suggested that patients could administer their own dialysis, considering this the highest level of patient participation.

*As a haemodialysis patient, you can of course participate in your own treatment. You can learn to cannulate the fistula, to set up the machine and to a certain extent run the dialysis.* (Participant D).

Other nephrologists referred to patient self-management as a way of decreasing health costs, because training patients in preparing the dialysis machine had the potential to save staff resources. Nephrologists and nurses typically discussed and assessed patient ability to perform self-care activities. They did not involve patients in this process, albeit the nurses encouraged participation from patients whom they had evaluated as able to participate, for instance, in self-cannulating. One of the nephrologists expressed: *It’s not appropriate for everyone to be trained and involved. But I think they should get the chance* (Participant H). From this perspective, patient participation was limited to people willing to exercise hands-on tasks.

Self-management included for the patient to adhere to food and fluid restrictions and administer medication as prescribed. Hence, the nephrologists emphasised thorough patient information. They argued that well-informed patients would be equipped with the rationale to act responsibly, associating responsible patients with better adherence and, hence, better treatment outcomes.

*They must be allowed to make informed choices. They must know why we do things and why they get this treatment, the point of coming here so frequently, the point of the medications.* (Participant F).

This illustrates how nephrologists provided patients with a personal responsibility for making choices about lifestyle and healthcare to support clinical treatment and accomplish treatment goals.

#### Negotiated values

Within hospital haemodialysis, divergent aims and interests could create tensions between patients and healthcare professionals. Shaping patient behaviour was experienced as necessary, though challenging, especially in patients of younger ages, as they wanted to live their life to the fullest. When patients did not adhere to restrictions, it could put them in danger of fluid overload or hyperkalaemia. The nephrologists told of experiences in which patients negotiated with respect to fluid removal, presenting a different opinion than the machine automatically programmed. Patient preferences were not always considered. As one of them put it:

*We don’t feel that all of them have enough knowledge about the treatment to have an opinion about it* [the fluid removal]*, so no, we don’t always discuss it with them* … (Participant F).

Despite nephrologists’ efforts to provide information as a means of increasing patient knowledge, some paradoxically doubted patient evaluations of their own treatment. This doubt allowed their professional expertise to override patients’ experiential knowledge. Similarly, although they saw patient engagement as positive, the nephrologists could limit the engagement by considering some patients to *decide too much themselves* (Participant E). ‘Deciding too much’ was associated with straying from the prescribed treatment. Other tensions could relate to patients who negotiated on their dialysis schedules by requesting to disclose sessions ahead of programmed time or changing days of treatment. The nephrologists recognised that adjusting hospital haemodialysis to employment or family life could be challenging for patients, and their experiences included patients who did not manage to reach their scheduled appointments for dialysis. This led to delays in the units’ workflow and was not welcomed.

Although paying attention to treatment schedules and biomedical quantifications, some nephrologists considered the clinical goals to be parts of a whole and emphasised that patient experiences should influence treatment adjustments. To tailor the treatment, they avoided being overly guided by biomedical targets and focused attention to listening to patients’ individual experiences of health and wellbeing between the dialysis sessions. One of them put it like this:

*Dialysis isn’t a pre-set treatment impossible to adjust to individual needs… many are unaware of that… but dialysis is about so much more than just prescribing a time-scaled treatment with this or that dialysate solution and this or that filter* [dialyzer membrane]. (Participant I).

Individual adjustments to treatment could decrease tensions, though the nephrologists had to balance adjustments with what they considered sufficient treatment. This illustrates the span nephrologists faced when they on the one hand aimed to provide evidence-based healthcare, while they on the other hand were expected to recognise patients’ preferences and values. When reflecting on their own practices, not all nephrologists considered patient experiences or issues without the potential for clinical adjustment to be their concern. For instance, some nephrologists saw hospital haemodialysis as a ‘take it or leave it’ treatment offer and preferred standardisation to individually customised treatment. Others experienced patient needs as never-ending and told of having to put up boundaries as to the issues with which they as nephrologists should engage and which issues they could leave to other professions.

#### Hindrances for patient participation

The nephrologists claimed that the increased focus provided by policy documents had made them more aware of patient participation. Some spoke of a shift in the role of physicians towards more person-centred care. Nevertheless, they considered the organisation of healthcare services an obstacle to accomplish patient participation. They experienced that the number of patients increased without additional resources being provided. Some suggested that the organisational system did not allow for true patient participation.

*You want to provide patient flexibility, but it is not always possible when you don’t have enough machines and there are cutbacks in addition and—it just makes it difficult.* (Participant A).

Efficiency focus was prominent in the clinics, and the nephrologists experienced time pressure as an inhibitor to involving patients. To ensure the accomplishment of everyday priorities, they considered it most effective to make the decisions themselves. Thus, their ideals of patient involvement suffered.

*If you are concerned with people and your patients, you should be concerned with patient participation as well. However, it takes more of your time. If you just decide on behalf of the patients, you get things done faster.* (Participant G).

According to the nephrologists, busy schedules could lead to loss of valuable information, for instance when patients did not want to bother healthcare professionals by conveying their symptoms. Thus, nephrologists could fail to notice negative treatment trends in the patients, such as fluid overload.

However, profoundly implemented ways of working could inhibit patient participation as well. For instance, nephrologists and nurses usually discussed the patient’s treatment in scheduled meetings without the patient’s presence. Nephrologists considered professionals’ meetings to be more effective compared with meetings that involved the patient, and typically informed patients about findings and treatment plans through ward rounds. Some nephrologists assessed it inappropriate to bring patients into professional meetings, as it would expose them to medical terms they would not understand and worry them for no purpose. To support patient privacy, confidential conversations between the nephrologist and the patient were arranged on the patient’s request.

## Discussion

The current study explored the experiences of nephrologists with patient participation for people of working ages in need of long-term dialysis. Following the ESRD trajectory, the nephrologists focused on individual choice when deciding on dialysis modality and emphasised that patients should make the final decision. We identified two approaches to accomplish this goal. In the first approach, nephrologists expected patients to make their choices based on information provided by healthcare professionals. In the second approach, they recognised patient values and preferences through shared decision-making. Within hospital haemodialysis, nephrologists associated patient participation with self-management, for instance, engaging patients in hands-on self-care activities. They identified tensions occurring from divergent patient–professional values and organisational structures as barriers to patient participation.

Nephrologists attending our study experienced the modality decision as complex but considered it important to let patients make the final choice. In westernised cultures, the ability to exercise choice is valued as an expression of autonomy [25]. Choice enables patients to direct their own course in accordance with individual preferences. However, it also leads to patient responsibility for their choices, and awareness of the possibility of making the wrong choice could be experienced as stressful [25]. Healthcare professionals may underestimate patients’ vulnerability and lowered capacity for decision-making when faced with illness [26]. This point to the delicate balance between involving patients in medical decisions without leaving them with a feeling of abandonment during the process [12].

Providing patients with well-balanced information about treatment options to enable them to choose the most suitable dialysis modality is in line with medical guidelines [6, 27]. However, people’s choices may differ depending on how the information is provided [28, 29]. Although focused on the individual’s freedom of choice, nephrologists in our study framed information about treatment options in a way that steered the patients towards a specific dialysis modality. Nephrology guidelines and health policy advocate a PD-first approach to increase home-based uptake [27]. This could lead nephrologists to, on the one hand downplay disadvantages of this treatment, or on the other hand favour the advantages of the treatment with which they are most familiar [30, 31]. By actively framing information, subjective interpretation is recognised as an integral part of the information [29]. Uneven knowledge in patient–professional relationships may favour medical preferences to achieve specific goals. Hence, patients may let the professional decide, believing their individual preferences are superfluous compared to medical expertise [32].

In the current study, nephrologists considered self-care activities equal to patient participation, seeing self-care as a source of patient empowerment. This is in line with a Swedish study [33] which found that healthcare professionals viewed the performance of dialysis as the ultimate form of patient participation in ESRD care. Patients, however, considered the choice of having the staff run their dialysis, at certain times or continuously, as an act of participation [33]. This is in accordance with a broader understanding of patient participation that includes dialogue, involvement in care, mutual shared knowledge and the management of self-care [34]. Patient participation is moreover contextual, allowing patients to move between different levels of participation [35]. The shifting of tasks from professionals to patients has potential to increase patient empowerment. Additionally, as suggested by some of the nephrologists in our study, task shifts may have economic outcomes as they can compensate for a shortage of healthcare providers [36].

Nephrologists in our study supported patient participation, which is in accordance with previous findings [20]. They nevertheless sustained traditional patient–professional approaches, for instance, by defining acceptable levels of patient participation, and excluding patients from discussions about treatment plans and self-care tasks. Healthcare teams typically consist of professionals, who may discuss treatment and set up care goals solely from their professional perspectives [37]. Including patients in team meetings may provide divergent and additional perspectives in accordance with patient-centred care [38]. By involving patients in decision making about treatment options and encourage them express their individual preferences and values, more patient-centred goals are possible [37]. Involving patients is a way to support patients’ capacity to restore autonomy and regain control of areas of their lives which are important to them [26, 38, 39]. It thus has potential to mitigate the patient-professional tensions occurring from divergent priorities, as experienced by nephrologists in the current study. However, patients entering the ESRD trajectory may not be aware of their legal right to participate [32, 40]. Nephrologists should therefore emphasise and encourage patient participation from their very first pre-dialytic patient meetings, as expressed by some of our study participants.

Some nephrologists did not trust their patients’ experiential knowledge. The complexity of ESRD patients [41] may induce nephrologists to emphasise evidence-based medical knowledge over patients’ experiential knowledge. They may thus prefer to make the decision themselves, and refrain from encouraging patient participation. Trust is an essential part of the patient–professional relationship, often directed from patients to the provider [42]. However, trust is reciprocal, which implies that professionals may also trust or mistrust their patients [43]. This reciprocity is evident in how patients being trusted by professionals may honour this trust by following medical advice [43]. Having a past disagreement with healthcare professionals, patients may be less likely to adhere to medical recommendations or rely on professional judgment. Rather, they are likely to take control and make medical decisions themselves [44].

Nephrologists attending this study recognised several existing factors that hampered patient participation. Diminishing these factors requires a broadened attention on individual and organisational levels as well as collaboration and commitment within the healthcare team. Nephrologists are not disentangled from a multidisciplinary approach, in which especially their alliances with dialysis nurses are important. Dialysis nurses are a key element in patient information as well as self-care management, care coordination, continuity, and exchange of health information [45]. A tidy collaboration between nephrologists and nurses that includes a common conceptual understanding thus has potential to promote patient participation. Lack of a collective understanding may result in practice approaches based on individual conceptualisations. This may in turn lead to a belief by professionals that they are facilitating patient participation even though they are practising within traditional frameworks [9, 20]. Heightening individual competence in shared decision-making—as well as team competence—enables patient–professional discussions that go beyond the giving of information and incorporate patients’ values and lifestyle preferences in joint treatment plans. On an organisational and political level, moving patient participation from ambition to reality is an ongoing activity. However, modern healthcare is complex and multifaceted, entailing organisational, political, and economic features that may be less compatible with the ideals of patient-centred care [18, 38]. For nephrologists, the requirement of promoting patient participation adds to several other requirements [46]. No incentives are included, however, and opting out of it has no consequences for healthcare professionals. It may therefore be easier and more effective for professionals to adhere to well-established clinical standards. Time as well as workloads associated with the complexity of ESRD patients require considerations when setting up budgets and allocating costs [41]. Ultimately, the responsibility for implementing patient participation lies at political and organisational levels, for instance, through optimising working conditions and educating staff.

### Strengths and limitations

The study sample consisted of ten nephrologists of various ages and gender who have their nephrology practice in Central Norway. We closed data collection when both variance and patterns appeared in the data. Although generalisation of the findings was not the intention, the issues presented in our study are legitimate and may be applicable to other contexts of haemodialysis. The first author has previously served several years as a haemodialysis nurse. Her extensive field knowledge was valuable in conducting the study. However, field knowledge comes with potential preconceived notions. Having co-authors from other backgrounds allowed for new interpretations of the findings. We conducted most interviews at the nephrologists’ workplaces, which implied disturbances. As the nephrologists treated patients of various ages, it could have been difficult for them to focus solely on working age patients. Thus, experiences with patient participation in other age groups than the one studied could have influenced their answers.

## Conclusion

Our study shows that nephrologists have different approaches to patient participation in different phases of the ESRD trajectory. Addressing individual understanding as well as organisational structures are important factors to increase patient participation in ESRD care. Shared decision making, in which patient values are balanced against biomedical treatment targets, allows for mutual agreement between patients and healthcare professionals concerning medical plans and minimises the potential for patient–professional tensions.

## Data Availability

De-identified data from the current study (audio-recordings, transcripts, and field notes) are being kept on a password-secured server. Written consents are being stored in a locked cabinet in the Møre og Romsdal Hospital Trust. The datasets used and analysed during the current study are available from the corresponding author on reasonable request.
